# Our Journal

**Published:** 1846-06

**Authors:** 


### PART IV.—EDITORIALS.

### ARTICLE X.

The first No. of the new series of our Journal, which should have been sent to our Subscribers the first of April last, was unavoidably delayed in its appearance till the first of May. This, the second No., we publish when it is due, the first of June, thus allowing but one month instead of two, as will be the case hereafter, to elapse from one issue to another.

Having had but this short time, therefore, to hear from our correspondents, and for the reception of new works and exchanges, we are unable to give to our readers, at this time, an amount of original matter and recent medical intelligence, equal to what we intend shall appear in subsequent numbers.

